# Introduction to NIRS International Open Laboratory (IOL)

**DOI:** 10.1093/jrr/rrt179

**Published:** 2014-03

**Authors:** Ryuichi Okayasu

**Affiliations:** International Open Laboratory, National Institute of Radiological Sciences, 4-9-1 Anagawa, Inage-ku, Chiba 263-8555, Japan

## Abstract

**Objectives of IOL:** We have two primary objectives: (i) To conduct world premier research in radiation sciences by collaborating with distinguished scientists from leading research institutes in the world. (ii) To promote further internationalization at NIRS by active research collaboration with foreign scientists.

**Brief history:** IOL started at the end of 2008 and its first term ended in March 2011after 2 years and four months. Drs Hirohiko Tsujii and Ohtsura Niwa were very instrumental in the establishment of IOL. The first term IOL has three unit leaders and three distinguished scientists: These are Yukio Uchihori for the Space Radiation Research unit working with Professor Tom Hei from Columbia University (USA), Takeshi Murakami for the Particle Therapy Model Research unit working with Professor Anders Brahme from Karolinska Institute (Sweden) and Ryuichi Okayasu for the Particle Radiation Molecular biology unit working with Professor Penny Jeggo, University of Sussex (UK). Dr Tsujii was named as Director of the first-term IOL. These three units scored ‘excellent’ for overall evaluation for their accomplishment by the international review committee organized in 2010. Many workshops were held by these IOL units and helped further the internationalization of NIRS.

**Current IOL:** In April 2011, the second-term IOL began and will complete in March 2014. As for the first-term IOL, each unit was selected by a special committee organized within NIRS. The structure of the second-term IOL is shown in Fig. 1. This time, four units and five distinguished foreign scientists were chosen; among them, Professors Jeggo, Brahme and Hei are continuing from the first term. Many exchanges of researchers have been arranged and further collaborative publications have been produced. In one of the IOL-sponsored meetings, we were able to invite renowned radiobiologist Professor Eric Hall from Columbia University; this conference attracted many people from all over Japan. Recognition of IOL has spread to international communities as sponsored activities have been mentioned in the websites of international organizations such as CERN (Europe), Chordoma Foundation (USA), Colorado State University (USA) and GSI (Germany). IOL activities also helped attract several external grants including Japanese KAKENHI grants. Many more activities including hosting international sessions in domestic meetings and sponsoring special seminars have taken place and will continue as long as the NIRS IOL system exists.

**Fig 1.**
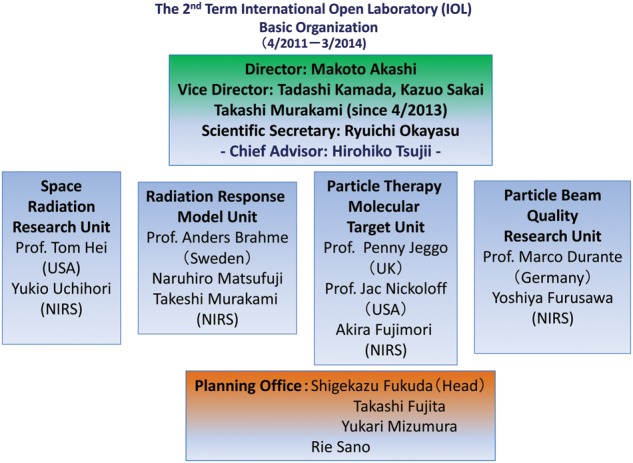
Structure of the second-term NIRS International Open Laboratory.

Structure of the second-term NIRS International Open Laboratory.

**Conclusion:** NIRS IOL significantly contributed to the internationalization of NIRS and is serving as a good model for international scientific collaboration.

